# Targeting Ferroptosis by Ubiquitin System Enzymes: A Potential Therapeutic Strategy in Cancer

**DOI:** 10.7150/ijbs.73790

**Published:** 2022-08-29

**Authors:** Yu Meng, Huiyan Sun, Yayun Li, Shuang Zhao, Juan Su, Furong Zeng, Guangtong Deng, Xiang Chen

**Affiliations:** 1 Department of Dermatology, Hunan Engineering Research Center of Skin Health and Disease, Hunan Key Laboratory of Skin Cancer and Psoriasis, Xiangya Hospital, Central South University, Changsha, Hunan, China.; 2 National Clinical Research Center for Geriatric Disorders, Xiangya Hospital, Central South University, Changsha, Hunan, China.; 3 Department of Oncology, Xiangya Hospital, Central South University, Changsha, Hunan, China.

**Keywords:** Ferroptosis, E3 ubiquitin ligase, Deubiquitinating enzyme, Cancer therapy

## Abstract

Ferroptosis is a novel type of regulated cell death driven by the excessive accumulation of iron-dependent lipid peroxidation. Therapy-resistant tumor cells, particularly those in the mesenchymal-like state and prone to metastasis, are highly susceptible to ferroptosis, suggesting that induction of ferroptosis in tumor cells is a promising strategy for cancer therapy. Although ferroptosis is regulated at various levels, ubiquitination is key to post-translational regulation of ferroptotic cell death. E3 ubiquitin ligases (E3s) and deubiquitinating enzymes (DUBs) are the most remarkable ubiquitin system enzymes, whose dysregulation accounts for the progression of multiple cancers. E3s are involved in the attachment of ubiquitin to substrates for their degradation, and this process is reversed by DUBs. Accumulating evidence has highlighted the important role of ubiquitin system enzymes in regulating the sensitivity of ferroptosis. Herein, we will portray the regulatory networks of ferroptosis mediated by E3s or DUBs and discuss opportunities and challenges for incorporating this regulation into cancer therapy.

## Introduction

Ferroptosis, a newly identified form of cell death, has attracted significant attention as a potential therapeutic target for cancer 1-3. Some therapy-resistant tumor cells, particularly those in the mesenchymal and dedifferentiated state, are vulnerable to ferroptosis inducers (FINs), which further underscores the potential of ferroptosis induction to eradicate tumors 4-7. Unique among other cell death modalities in morphological and biochemical features, ferroptosis is characterized by shrunken mitochondria, increased mitochondrial membrane density, iron dependence, accumulation of reactive oxygen species (ROS) and lethal lipid peroxidation^8^, ^9^. Recently, the regulation of ferroptosis at the epigenetic, transcriptional, and post-transcriptional levels has been extensively studied and reviewed 10-13. However, the post-translational regulation of ferroptosis has rarely been summarized and remains largely unknown.

Ubiquitination, a process that covalently attaches ubiquitin, is one of the most well-studied regulatory post-translational modifications 14. It is tightly coupled to the regulation of protein interactions, trafficking, degradation, and enzymatic activities, thereby affecting a wide spectrum of cellular processes, including ferroptosis, lysosomal degradation, DNA repair, apoptosis, and other diverse processes 15-17. Similar to other cell modifications, the ubiquitin system is strikingly reversible, with the combined activity of ubiquitin conjugation counterbalanced by deconjugation. Ubiquitin conjugation is mediated by an enzymatic cascade (E1-E2-E3) that culminates in the activity of E3 ubiquitin ligases (E3s), which transfer ubiquitin to substrates for subsequent degradation via the 26S proteasome system^18^, ^19^. Conjugation can be reversed by deubiquitinating enzymes (DUBs), which cleave ubiquitin from substrate proteins and edit ubiquitin chains 20. Various E3s and DUBs have been implicated in tumorigenesis and progression by regulating oncogenes and tumor suppressors including such characteristics as stability, localization, and activation 21-23. Accumulating evidence indicates that ubiquitin system enzymes or their complexes regulate the susceptibility of cancer cells to ferroptosis, mainly by regulating ubiquitination and the protein stability of key regulators of ferroptosis 24-26. Therefore, a more comprehensive understanding of the ubiquitin system involved in the regulation of ferroptosis may provide new insights into cancer therapeutics.

With rapid advances in research on ferroptosis, it is important to summarize the latest studies on the regulation of ferroptosis via ubiquitin system enzymes. In this review, we outline the mechanism of ferroptosis and its dual role in cancer progression. We also thoroughly discuss the current understanding of the regulatory networks of ferroptosis mediated by E3s and DUBs and discuss the opportunities and challenges for incorporating this regulation into cancer therapy.

## The mechanism of ferroptosis

**Figure 1:**
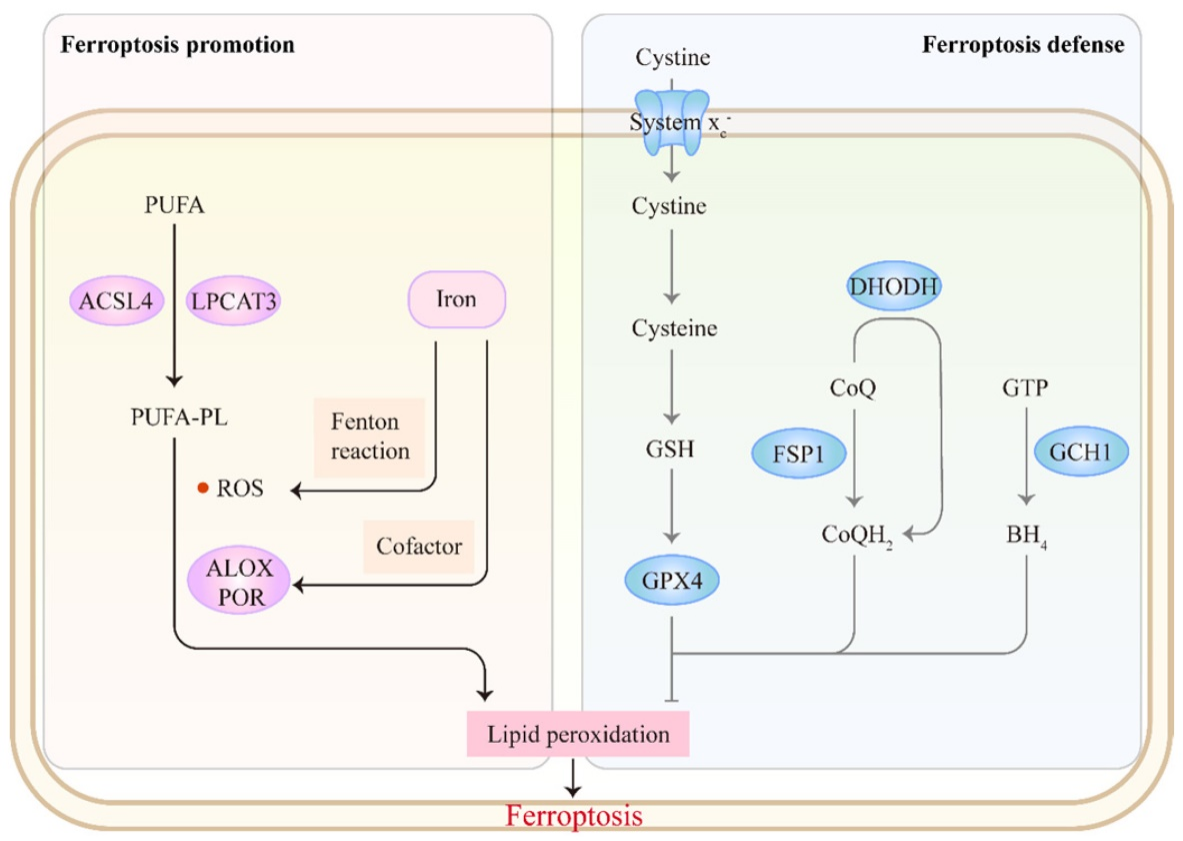
** The mechanism of ferroptosis.** Ferroptosis involves an antagonism between ferroptosis promotion and defense. The synthesis of PUFA-PLs by ACSL4 and LPCAT3 offers prerequisites for subsequent peroxidation. Excess iron promotes peroxidation of PUFA-PLs through two main mechanisms: 1) the generation of ROS by Fenton reaction and 2) the activation of lipoxygenases (ALOXs) or cytochrome P450 oxidoreductase (POR) as a cofactor, which favors ferroptosis onset. Correspondingly, cells have evolved ferroptosis defense systems against peroxidation damage: system x_c_^-^-GSH-GPX4, FSP1-CoQH_2_, DHODH-CoQH_2_ and GCH1-BH_4_ pathways.

The core mechanism of ferroptosis involves antagonism between ferroptosis promotion and defense (**Figure 1**). Ferroptosis is mainly driven by the unrestricted peroxidation of polyunsaturated fatty acid-containing phospholipids (PUFA-PLs) in cellular membranes 9. Incorporation of PUFAs into membrane PLs requires the enzymes acyl-CoA synthetase long-chain family member 4 (ACSL4) and lysophosphatidylcholine acyltransferase 3^27^, ^28^. Subsequently, excess iron promotes peroxidation of PUFA-PLs through two main mechanisms: 1) the generation of ROS by iron-mediated Fenton reaction and 2) the activation of iron-dependent lipoxygenases or cytochrome P450 oxidoreductases, which favor membrane damage and ferroptosis onset 29, 30. Correspondingly, cells have evolved ferroptosis defense systems against peroxidation damage. The system x_c_^-^-reduced glutathione (GSH)-glutathione peroxidase 4 (GPX4) pathway plays a central role in detoxifying lipid peroxidation 31, 32. Several non-GPX4 pathways, including FSP1-CoQH_2_, DHODH-CoQH_2_ and GCH1-BH_4_, also inhibit ferroptosis 33-37. Accumulating evidence indicates that targeting these ferroptosis-promoting or ferroptosis defense mechanisms could affect tumor behavior and treatment. We refer readers to other reviews for a detailed discussion of the mechanisms of ferroptosis 13, 32, 38-40.

## Ferroptosis involved in cancer

**Figure 2:**
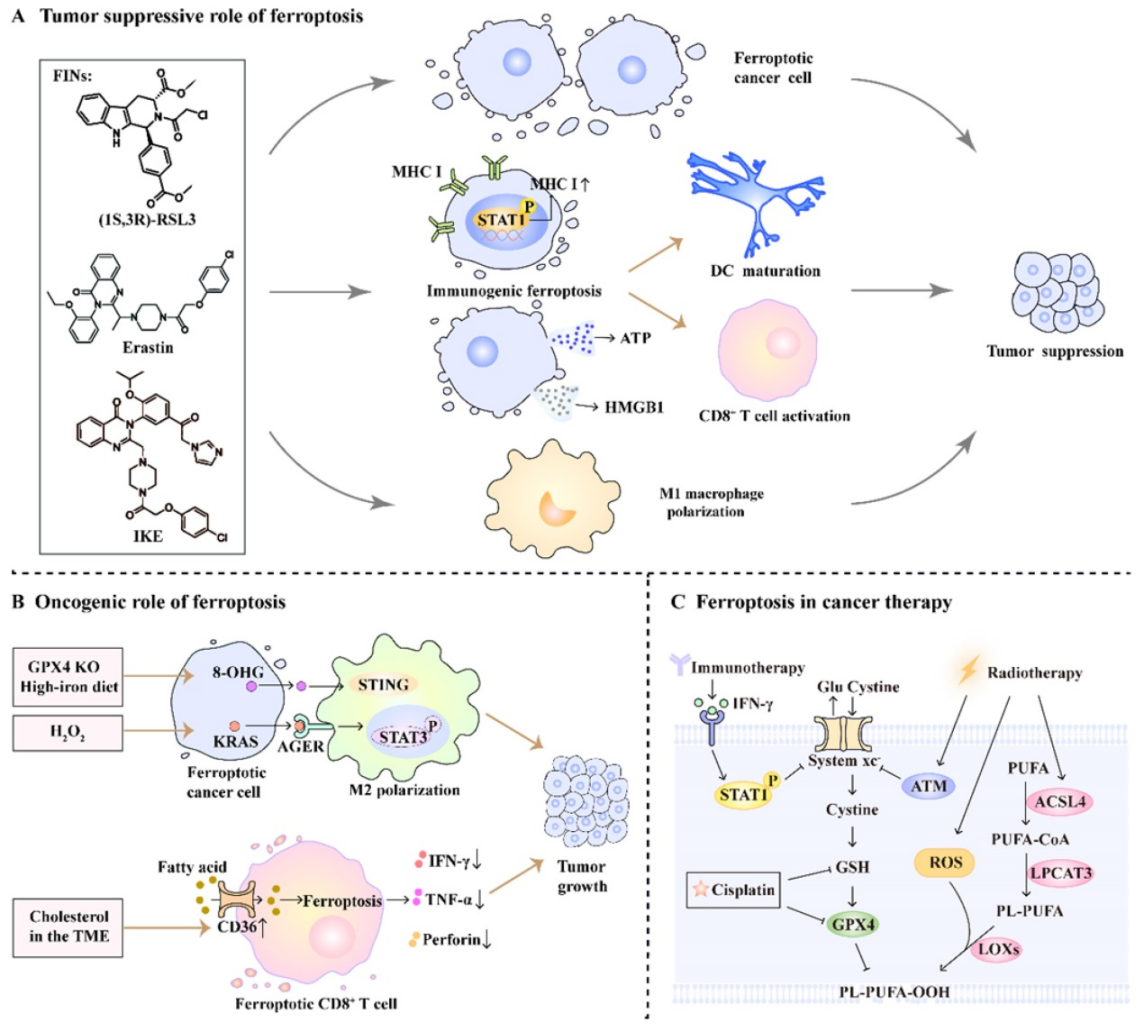
** The role of ferroptosis in cancer progression and therapy.** (A) FINs such as (1S,3R)-RSL3, erastin, and imidazole ketone erastin (IKE)-induced ferroptotic damage has a tumor suppressive role. On one hand, FINs can directly induce ferroptosis in cancer cells. On the other hand, ferroptosis induction promotes DC maturation, primes CD8^+^ T cell activation and facilitates M1 macrophage polarization. (B) Ferroptosis has an oncogenic role by promoting M2 macrophage polarization and impairing CD8^+^ T cell function. (C) Radio-, chemo-, and immunotherapy can trigger ferroptosis by affecting ferroptosis-related pathways.

Ferroptosis is well known for its anti-tumor effects, and its pro-tumor role has recently been reported. Here, we provide a thorough and critical analysis of the dual role of ferroptosis in cancer progression, as well as its synergistic effect on cancer therapy (**Figure 2**).

### The tumor suppressive role of ferroptosis

Ferroptosis induction is an effective method for killing cancer cells through the genetic or pharmacological intervention of ferroptosis-related pathways. Ferroptosis can be induced by genetic ablation of solute carrier family 7 member 11 (SLC7A11) or GPX4 in cancer cells, leading to significant tumor suppression 7, 41. Additionally, several clinically approved drugs and experimental small-molecule compounds (sulfasalazine, sorafenib, erastin, and its analog imidazole ketone erastin) are well-known inducers of ferroptotic cancer cell death through the inhibition of system x_c_^-^42-46. Altretamine and (1S,3R)-RSL3 also show antineoplastic activity and provoke ferroptosis via inhibiting GPX4 directly 31, 45, 47.

The induction of ferroptosis reshapes the tumor microenvironment (TME) to stimulate an anti-tumor immune response, thereby suppressing tumor progression. Ferroptosis induction increases cancer cell immunogenicity by upregulating MHC class I expression and releasing damage-associated molecular patterns (DAMPs; e.g., ATP and HMGB1) 48, 49. On the other hand, ferroptotic stress promotes metabolic reprogramming and pro-inflammatory signaling activation in tumor-associated macrophages (TAMs), resulting in an antitumoral M1-like phenotype. This polarization induces phagocytic killing activity and inhibits tumor metastasis 50. These findings suggest that the induction of ferroptosis could affect anti-tumor immunity and inhibit tumor progression.

### The oncogenic role of ferroptosis

However, emerging studies have also suggested that ferroptosis plays an oncogenic role in tumor immunity. DAMPs released from ferroptotic cancer cells, such as 8-OHG and KRAS^G12D^, contribute to macrophage-induced tumorigenesis. Ferroptotic damage triggered by high-iron diets or GPX4 knockout facilitates the release of 8-OHG and activation of the STING-dependent DNA sensor pathway, which results in TAM infiltration and pro-tumorigenic M2 polarization during Kras-driven pancreatic ductal adenocarcinoma (PDAC) in mice 51. Similarly, exosomes packaged with the DAMP KRAS^G12D^ are released from PDAC cells during hydrogen peroxide-elicited ferroptosis and are taken up by macrophages via advanced glycosylation end product-specific receptor (AGER). This uptake causes AGER-mediated STAT3 activation, leading to M2 macrophage polarization and PDAC growth 52.

Inducing ferroptosis in CD8^+^ T cells effectively limits their anti-tumor activity and more importantly, supports tumor growth. Ma et al. found that TME cholesterol-induced CD36 expression in tumor-infiltrating CD8^+^ T cells is associated with tumor progression and poor survival in human and murine cancers 53. Mechanistically, the membrane glycoprotein CD36 mediates fatty acid uptake to increase arachidonic acid levels, which promotes lipid peroxidation and ferroptosis in CD8^+^ T cells 53. These findings highlight the dual role of ferroptosis in tumor progression, depending on the cellular context.

### The role of ferroptosis in tumor treatment

Considering the anti-tumor role of ferroptosis, targeting ferroptosis is a promising strategy for cancer therapy. Intriguingly, radio-, chemo-, and immunotherapies have been reported to induce ferroptosis; boosting ferroptosis with FINs could further potentiate their efficacy. Ionizing radiation triggers ferroptosis by inhibiting SLC7A11 expression, upregulating ACSL4, and inducing ROS production 54, 55. FINs also render cancer cells more sensitive to radiation and improve efficacy of radiotherapy 55, 56. Chemotherapeutic agents such as cisplatin induce ferroptosis via depletion of GSH 57. Treatment with FINs potentiates the anti-tumor effect of chemotherapeutic agents such as cisplatin, gemcitabine, doxorubicin, and cytarabine 41, 57-59. In addition, interferon-gamma released from immunotherapy-activated CD8^+^ T cells triggers ferroptosis in tumor cells by inhibiting system x_c_^-^ expression 60. FINs in combination with checkpoint blockades synergistically enhance T-cell-mediated anti-tumor immunity and induce ferroptosis in tumor cells 48, 61. Furthermore, several clinical trials are underway to assess the safety and efficacy of the combination of FINs with traditional therapies in patients with cancer (NCT04205357, NCT03247088). Taken together, FINs have great potential in tumor therapy, especially in combination with conventional therapies.

## Ferroptosis regulated by E3s

**Figure 3:**
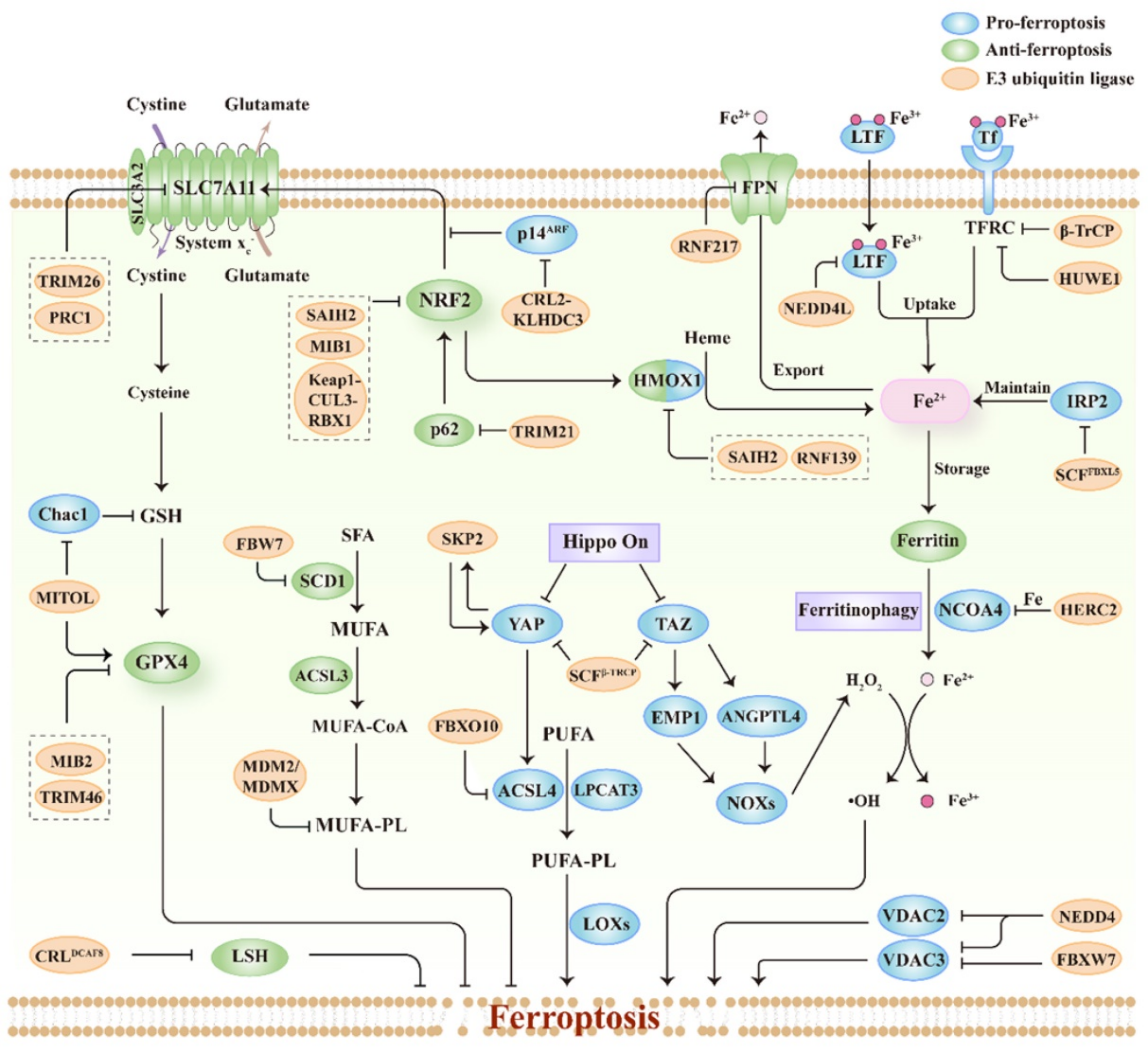
** The relationship between ferroptosis regulators and E3s.** The system x_c_^-^-GSH-GPX4 pathway is important for protecting cells from ferroptosis and E3s participate in regulating this pathway. E3s can regulate lipid metabolism pathways including polyunsaturated fatty acid-containing phospholipids (PUFA-PL) and monounsaturated fatty acid-containing phospholipids (MUFA-PL). E3s also regulate key factors associated with the uptake, export, storage and utilization of iron during ferroptosis. Besides, E3s are involved in the modulation of other ferroptosis regulators including NRF2, Hippo pathway effectors (YAP and TAZ), VDAC, and LSH.

E3s include three major classes based on their domains and manners of ubiquitin ligation: really interesting new gene (RING), homologous to the E6-AP carboxyl terminus (HECT), and RING1-between-RING2 (RBR). E3s mainly target ferroptosis drivers or suppressors for ubiquitination, and regulate their degradation, localization, or transcriptional activity, thus modulating cellular sensitivity to ferroptosis. Herein, we summarize the studies on ferroptosis regulated by E3s published to date (**Figure 3; Table 1**). Importantly, we also discuss the relationship between ferroptosis-associated regulators and E3s (**Figure 3; Table 1**), thereby providing potential for the discovery of new E3s responsible for regulating ferroptosis.

### Regulation of system x_c_^-^-GSH-GPX4 axis

System x_c_^-^ is a heterodimeric complex composed of SLC7A11 and SLC3A2, which functions as a cystine/glutamate antiporter on the cell surface 62. Cystine uptake mediated by system x_c_^-^ promotes the biosynthesis of GSH 63. Accordingly, the inhibition of system x_c_^-^ causes GSH depletion and GPX4 inactivation, contributing to ferroptosis induction 13. E3s have been reported to regulate protein stability and gene transcription of SLC7A11 via ubiquitination. TRIM26, a member of the RING-type E3s, mediates the ubiquitination and proteasomal degradation of SLC7A11, inducing ferroptosis in hepatic stellate cells 64. Histone 2A ubiquitination is associated with the regulation of gene transcription, including SLC7A11 65, 66. Polycomb repressive complex 1 is an E3 ligase responsible for ubiquitinating histone 2A and downregulating SLC7A11 expression by increasing ubiquitination of histone 2A on the SLC7A11 promoter in human renal cancer cells 66. However, the role of polycomb repressive complex 1-mediated transcriptional repression of SLC7A11 in ferroptosis and tumor development awaits further investigation.

GSH, the cofactor of GPX4, helps GPX4 detoxify phospholipid hydroperoxides 67. The depletion of GSH can inactive GPX4 and trigger ferroptosis 68. ChaC glutathione-specific γ-glutamylcyclotransferase 1 (Chac1) is responsible for degradation of GSH and regulates the sensitivity of cancer cells to ferroptosis 69, 70. Recent studies have demonstrated that Chac1 is inhibited by mitochondrial ubiquitin ligase (MITOL), a mitochondrial membrane-associated E3 ligase, via the eIF2a-ATF4 pathway in cardiomyocytes 71. Consistently, downregulation of MITOL increased Chac1 expression, resulting in decreased GSH and GPX4 levels. Therefore, MITOL knockdown promotes lipid peroxidation and ferroptosis by disrupting GSH homeostasis and GPX4 expression 71.

GPX4 is a crucial enzyme that directly catalyzes the reduction of phospholipid hydroperoxides to protect membranes from ferroptotic damage 72. Several E3 ligases interact with GPX4 and mediate its degradation. TRIM46 is upregulated by elevated glucose in a time-dependent manner, which interacts with GPX4 and facilitates its ubiquitination and subsequent degradation 73. Therefore, the upregulation of TRIM46 contributes to high glucose-induced ferroptosis and growth inhibition of human retinal capillary endothelial cells. Furthermore, sevoflurane administration induces E3 ligase mind bomb 2 (MIB2) expression, thus promoting ferroptosis in hippocampal neurons. Mechanistically, MIB2 binds to GPX4 and mediates ubiquitination of GPX4 74. Collectively, the activation of TRIM46 or MIB2 expression under different stimuli promotes GPX4 ubiquitination and ferroptosis in certain cellular contexts. However, the mechanism by which TRIM46 and MIB2 mediate the ubiquitination of GPX4 remains to be explored.

### Regulation of iron metabolism

Iron plays a key role in ferroptosis. Cellular iron levels are tightly regulated by iron metabolism, including iron uptake, storage, utilization, and export 75. Recently, the link between iron metabolism and E3s has been extensively investigated.

Transferrin is a carrier protein loaded with ferric ions and can be imported into cells via transferrin receptor (TFRC)-mediated endocytosis 76. This non-heme iron uptake is necessary for ferroptosis, because downregulation of TFRC suppresses ferroptosis induced by cystine starvation or (1S,3R)-RSL3 treatment 77, 78. The E3 ligase β-TrCP promotes ubiquitination and degradation of TFRC via the adaptor protein TRIB2, decreases labile iron levels in liver cancer cells, and confers resistance to (1S,3R)-RSL3- or erastin-induced ferroptosis 24. Overexpression of β-TrCP inhibits human liver cancer cell proliferation, possibly by reducing iron (which is essential for tumor cell growth). Similarly, in hepatocytes, the HECT-type E3 ligase HUWE1 has been reported to target TFRC for ubiquitination and proteasomal degradation, thereby regulating iron metabolism and inhibiting ferroptosis 79. Lactotransferrin, also known as lactoferrin, is an iron-binding protein that belongs to the transferrin family. The HECT-type E3 ligase NEDD4L suppresses ferroptosis by promoting the degradation of lactotransferrin and decreasing intracellular iron accumulation in human pancreatic and ovarian cancer cells 80. Therefore, knockdown of NEDD4L enhances anti-tumor activity triggered by (1S,3R)-RSL3 or erastin.

Heme oxygenase-1 (HMOX1) catalyzes heme into ferrous iron in cells and promotes ferroptosis by increasing iron load 81. Zinc protoporphyrin, a HMOX1 inhibitor, prevents erastin-induced ferroptosis, whereas hemin, an HMOX1 inducer, promotes erastin-induced ferroptosis. Consistently, HMOX1 knockout has been found to suppress ferroptosis, whereas HMOX1 overexpression accelerates ferroptosis in HT-1080 fibrosarcoma cells 82. These findings were consistent in the heart, but not in renal proximal tubular cells or liver cancer cells 83-85. Absentia homolog 2 (SIAH2), a member of the RING-type E3s, has been reported to regulate HMOX1 both at the transcriptional and post-translational levels 85, 86. SIAH2 downregulates HMOX1 expression by destabilizing nuclear factor erythroid 2-related factor 2 (NRF2) that functions as a transcription factor of HMOX1 86. SIAH2 decreases HOXM1 protein levels by promoting proteasomal degradation of HMOX1 in specific organs such as the heart 85. In addition, knockout of SIAH2 increases sensitivity to ferroptosis triggered by erastin or (1S,3R)-RSL3 treatment, which is possibly due to the higher proferroptotic HMOX1 level 85. In addition to SAIH2, E3 ligase RNF139 interacts with HMOX1 and mediates its ubiquitination and degradation, which contributes to the tumor-suppressive effect of RNF139 in renal carcinoma cells 87.

Ferritin, the main iron storage protein, is composed of ferritin heavy chain 1 and ferritin light chain 88. Ferritin can be degraded by autophagy receptor nuclear receptor coactivator 4 (NCOA4)-mediated ferritinophagy 89. Recent studies suggest that ferritinophagy is involved in triggering labile iron overload and promoting ferroptosis 90, 91. Intriguingly, NCOA4-mediated ferritinophagy can be regulated by E3s, and this regulation is dependent on iron. When cellular iron levels are high, the C-terminal domain of NCOA4 binds to iron. HECT-type E3 HERC2 interacts with iron-bound NCOA4 and accelerates the degradation of NCOA4 via the ubiquitin-proteasome system 92. In the absence of iron, HERC2 separates from iron-free NCOA4 to stabilize NCOA4 and promotes ferritinophagy 92. Therefore, HERC2 plays an important role in the iron-dependent turnover of NCOA4, but further investigation is required to establish whether this regulation affects cellular sensitivity to ferroptosis, especially in cancer cells.

Ferroportin (FPN) is the only iron exporter protein found in mammalian cells 93. FPN inhibits erastin-induced ferroptosis via decreasing labile iron pools in cells 94, 95. RNF217 acts as a novel member of the RBR-type E3s and regulates iron homeostasis through its E3 ubiquitin ligase activity by modulating FPN degradation in macrophages 96. The direct role of RNF217-mediated ubiquitination and degradation of FPN in ferroptosis requires further investigation. Iron regulatory protein 2 (IRP2) is an RNA-binding protein that maintains iron homeostasis through post-transcriptional regulation of TFRC, ferritin, and FPN 97, 98. Several studies have shown that IRP2 is a driver of ferroptosis 99, 100. F-box and leucine-rich repeat protein 5 (FBXL5), the substrate recognition component of the Skp1-Cul1-F-box (SCF) E3 ligase complex, recognizes IRP2 and contributes to the proteasomal degradation of IRP2 with sufficient oxygen and iron in eukaryotes 101, 102. Similarly, FBXL5 deficiency leads to cellular iron overload via decreased FBXL5-mediated degradation of IRP2, which promotes ROS production in hematopoietic stem cells 103. However, the potential role of FBXL5 in ferroptosis and whether other E3s are involved in iron metabolism remain unclear.

### Regulation of lipid metabolism

E3 ligases can mediate the degradation and transcriptional regulation of enzymes involved in lipid metabolism, which is correlated with cellular sensitivity to ferroptosis. ACSL4 is an enzyme that contributes to the biosynthesis of PUFA-PLs and promotes lipid peroxidation and ferroptosis induced by GPX4 inhibition 27, 104. F-box-only protein 10 (FBXO10), a component of the SCF E3 complex, is responsible for substrate recognition. FBXO10 interacts with ACSL4 and promotes ACSL4 ubiquitination and degradation, which may alleviate ferroptosis induced by traumatic brain injury 105. In addition, stearoyl-CoA desaturase (SCD1) is a lipid-modifying enzyme that promotes monounsaturated fatty acid synthesis and protects cancer cells from ferroptosis 106. F-box and WD repeat domain-containing 7 (FBW7) is a subunit of the SCF E3 complex that is responsible for substrate recognition. Significantly, FBW7 represses SCD1 transcription by inhibiting the binding of NR4A1 and the SCD1 promoter and promotes lipid peroxidation and ferroptosis in pancreatic cancer cells 107. Consistently, FBW7 potentiates the tumoricidal effect of gemcitabine by promoting ferroptosis in pancreatic cancer. Furthermore, murine double minute 2 (MDM2) is an E3 ligase for ubiquitinating p53 108. Murine double minute X (MDMX) can form a heterodimer with MDM2, thereby enhancing the ligase activity of MDM2 109. Venkatesh et al. found that MDM2 and MDMX promote ferroptosis in a manner that is independent of p53 110. The MDM2-MDMX complex regulates lipids by altering PPARα activity and PPARα-mediated lipid remodeling in several tumor cell lines, especially in patient-derived glioblastoma cell lines 110. However, the key downstream genes of PPARα responsible for ferroptosis require further investigation.

### Regulation of NRF2

NRF2 functions as a transcription factor and activates multiple antioxidant genes, which mediates cellular antioxidant responses and limits lipid peroxidation and ferroptosis 83, 111, 112. The E3 ligase complex composed of Kelch-like ECH-associated protein 1 (Keap1), Cullin3, and RING-box protein 1 mediates ubiquitination and rapid degradation of NRF2 under quiescent conditions 113. However, under oxidative stress, cysteine residues of Keap1 are modified, leading to inactivation of the aforementioned ligase complex and subsequent NRF2 stabilization 114. In addition, sequestosome 1, also known as p62, competes with NRF2 for Keap1 binding, resulting in Nrf2 release and stabilization 114, 115. The E3 ligase TRIM21 binds to p62 via the PRYSPRY domain and ubiquitylates p62 at K7 via K63-linked ubiquitination 116. The ubiquitination of p62 by TRIM21 counteracts p62-mediated NRF2 activation 116. Consistently, downregulation of TRIM21 promotes antioxidant response and suppresses ferroptosis induced by doxorubicin 117. These findings suggest that E3s play an important role in redox homeostasis as well as ferroptosis owing to their regulation of the p62-Keap1-NRF2 pathway.

In addition, E3s can regulate the p14^ARF^-NRF2-SLC7A11 pathway to modulate ferroptosis. ARF, known as p14 alternate reading frame (p14^ARF^) in humans, inhibits NRF2 acetylation and NRF2-mediated transcriptional activation of SLC7A11^118^. KLHDC3 is an adaptor protein of Cullin-RING ligase (CRL) 2 that interacts with the C-terminal degron of p14^ARF^, triggering proteasomal degradation of p14^ARF^ in multiple cancer cell lines 119. Accordingly, the CRL2-KLHDC3 E3 complex mitigates the p14^ARF^-mediated inhibition of SLC7A11 transcription and suppresses ferroptosis, thus contributing to the pro-tumorigenic role of KLHDC3 119. Moreover, E3 mind bomb 1 promotes the ubiquitination and degradation of NRF2 via the proteasome pathway, which sensitizes lung cancer cells to ferroptosis 25. Collectively, dysregulation of NRF2 can lead to transcriptional changes and disturbed redox equilibrium, thereby regulating ferroptosis.

### Regulation of VDAC2 and VDAC3

The voltage-dependent anion channel (VDAC) is a channel that mediates metabolite and ion exchange across the outer mitochondrial membrane 120. The classic FIN, erastin, binds to VDAC and regulates its opening for ferroptosis induction 121. Current evidence has implicated that the degradation of VDAC2 and VDAC3 by E3s inhibits erastin-induced ferroptosis. FBXW7 acts as a substrate recognition component of the SCF E3 complex and mediates ubiquitination and degradation of VDAC3, conferring resistance to erastin-induced ferroptosis in acute lymphoblastic leukemia cells 122. In addition, E3 NEDD4 interacts with the PPxY motifs of VDAC2 and VDAC3 via its WW domain, thereby mediating K48-linked ubiquitination and degradation of VDAC2 and VDAC3 in melanoma cells 26. Interestingly, erastin treatment stimulates NEDD4 expression, which in turn NEDD4 inhibits erastin-induced ferroptosis via the degradation of VDAC2 and VDAC3. Therefore, this negative feedback regulation may provide new insights into how cancer cells maintain homeostasis by E3s in response to FINs 26.

### Regulation of Hippo pathway

The Hippo pathway regulates ferroptosis depending on cell density, a process in which E3s may also be involved. Under high density, Hippo signaling is activated and its downstream effectors, Yes-associated protein (YAP) and transcriptional coactivator with PDZ-binding motif (TAZ), are phosphorylated by LATS kinases, which promote YAP/TAZ cytoplasmic localization and prime them for further phosphorylation by casein kinase 1 123, 124. The SCF^β-TrCP^ E3 complex recognizes phosphorylated YAP/TAZ, leading to their ubiquitination and proteasomal degradation 125, 126. YAP and TAZ are transcriptional co-activators of TEAD, which upregulates some ferroptosis-promoting genes, including ACSL4, TFRC, ANGPTL4, and EMP1 127-129. Therefore, degradation and cytoplasmic sequestration of YAP/TAZ represses the expression of these ferroptosis drivers, thus leading to inhibition of ferroptosis. Additionally, E3s can regulate YAP localization. The SCF^SKP2^ E3 complex mediates K63-linked polyubiquitination of YAP, which increases its nuclear localization and transcriptional activity 130. Interestingly, SKP2 is one of transcriptional targets of YAP and sensitizes cancer cells to erastin-induced ferroptosis 131. As a result, a positive feedback loop may exist between YAP and SKP2, possibly regulating the sensitivity of cancer cells to ferroptosis. SKP2 also targets the transcription of threonine tyrosine kinase and TFRC, both of which partly contribute to SKP2-mediated ferroptosis 131.

### Regulation of epigenetic mechanism

Recent studies have shown that epigenetic regulation is also involved in ferroptosis. Lymphoid-specific helicase (LSH), also known as HELLS, is a chromatin-remodeling ATPases 132. LSH has been reported to suppress ferroptosis via epigenetic and transcriptional regulation of ferroptosis-related genes 133. E3s can control the protein stability of LSH to regulate epigenetic dynamics in ferroptosis. DCAF8 and WDR76 are potential substrate recognition receptors of CRL4, a member of the CRL family. CRL4^DCAF8^ targets the proteasomal degradation of LSH as an E3 complex, whereas WDR76 competitively inhibits this degradation process by hindering the assembly of the holo-CRL4^DCAF8^-LSH complex 134. Hence, this opposing regulatory strategy of CRL4^DCAF8^ and WDR76 controls the sensitivity of cancer cells to ferroptosis.

## Ferroptosis regulated by DUBs

**Figure 4:**
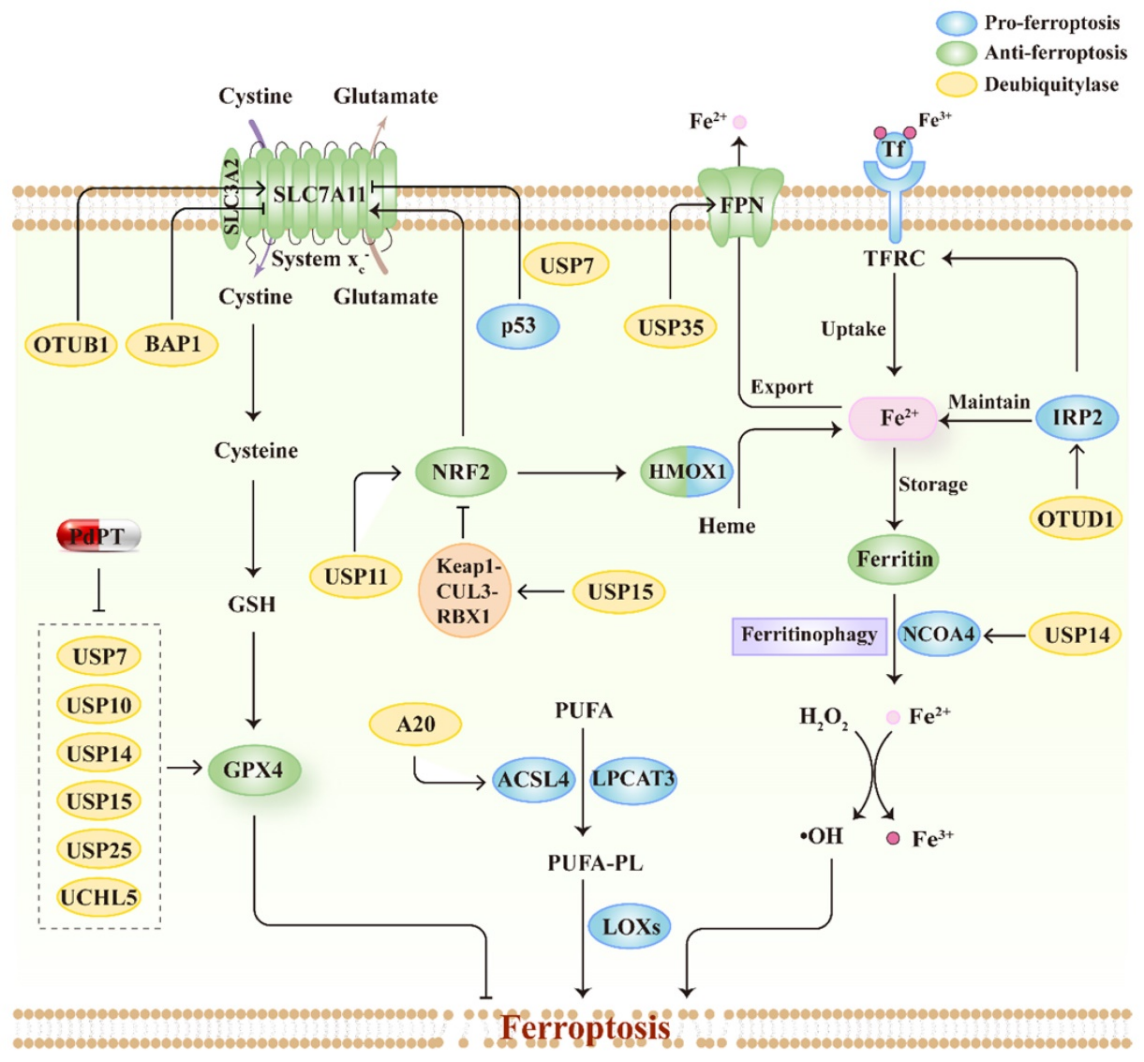
** The relationship between ferroptosis regulators and DUBs.** Schematic overview of DUBs involved in the process of ferroptosis by regulating multiple signal pathways, including system x_c_^-^-GSH-GPX4 pathway, lipid metabolism pathways and iron metabolism pathway. Moreover, PdPT, a pan-DUB inhibitor, could decrease GPX4 expression through inhibiting various DUBs.

DUBs are enzymes that functionally cut off ubiquitin chains from specific subsets of proteins. DUBs can be divided into seven superfamilies, including one metalloprotease superfamily, the Jad1/Pad/Mpn domain-containing metalloenzymes (JAMMs), and six cysteine-based superfamilies, the ubiquitin-specific proteases (USPs), ovarian tumor proteases (OTUs), Machado-Joseph disease domain proteases (MJDs), Ub C-terminal hydrolases (UCHs), motif interacting with Ub-containing novel DUB family (MINDYs), and the zinc-finger and UFSP domain protein (ZUFSP)^135^. The various structural elements of DUBs serve diverse biofunctions, including processing ubiquitin precursors and cleaving poly-ubiquitin chains to maintain free ubiquitin pools, stabilizing protein substrates from degradation via trimming ubiquitin chains, and editing ubiquitin chains to convert ubiquitin signals^136^. Similar to E3s, recent genetic studies have reported that DUBs regulate ferroptosis by modulating the substrate stability and signal transduction. Here, we summarize the mechanisms by which DUBs regulate ferroptosis and emphasize their roles in connecting ferroptosis to tumor suppression, thus further exploring the advantages of therapeutic targeting of DUBs in cancer treatment (**Figure 4; Table 2**).

### Regulation of system x_c_^-^-GSH-GPX4 axis

It appears that DUBs could modulate SLC7A11 expression in line with E3s and further influence tumor progression by inducing ferroptosis. BRCA1-associated protein 1 (BAP1), the first reported DUB that regulates SLC7A11, decreases the expression of SLC7A11 by deubiquitinating H2Aub and reducing its occupancy on SLC7A11 promoters 137. BAP1 triggers ferroptosis in multiple cancer cell lines, including kidney clear cell carcinoma, kidney papillary cell carcinoma, uveal melanoma, pheochromocytoma and paraganglioma, and invasive breast carcinoma 137, 138. Interestingly, BAP1 and E3 ligase PRC1 have opposite effects on H2Aub, but have the same effect on SLC7A11 expression, suggesting a coordinated function of ubiquitinase and deubiquitinase in ferroptosis regulation 138. USP7 nuclear translocation is promoted by p53, which leads to the deubiquitination of H2Bub1, thus decreasing SLC7A11 expression and activity in lung cancer during ferroptosis induction 139. OTUB1, an OTU family member deubiquitinase, has been previously reported to interact with SLC7A11 and regulate its stability. Rather than depending on its deubiquitination ability, OTUB1 stabilizes SLC7A11 by inhibiting the E2-conjugating enzymes recruited by E3 ligases 140. Accumulating evidence has shown that depletion of OTUB1 sensitizes multiple tumor cells to ferrotopsis, including glioma 141, colon cancer 142, breast cancer 143, and pancreatic cancer 144, thereby modulating OTUB1 might be a promising strategy for cancer therapy. Palladium pyrithione complex (PdPT) is a pan-inhibitor of multiple DUBs, including USP7, USP10, USP14, USP15, USP25, and UCHL5, which contributes to ferroptosis and apoptosis in NSCLC cell lines. Mechanistically, PdPT increases GPX4 ubiquitination and promotes its degradation by inhibiting DUBs activity 145. Overall, DUBs that regulate SLC7A11 are complicated in tumor cells. DUBs can enhance SLC7A11 stability by inhibiting its degradation, whereas other DUBs can decrease SLC7A11 expression through histone modification.

### Regulation of iron metabolism

Given the central role of iron in ferroptosis, it is unsurprising that DUBs are involved in the regulation of ferroptosis by modulating iron homeostasis. OTUD1 increases ferroptosis in colon cancer by stabilizing IRP2 and promoting iron uptake through TFRC 146. In contrast, USP35 stabilizes FPN and increases FPN-dependent iron export, thus inhibiting erastin- or RSL3-triggered ferroptosis and tumor-suppressive effects in lung cancer 94. USP14 was reported as a regulator of NCOA4, indicating the key role of USP14 in autophagy-dependent ferroptosis 147. In addition, inhibition of USP14 by 6-Gingerol is accompanied with the changed expression of NCOA4 and ferritin heavy chain 1 and accordingly increases ferroptosis 148.

### Regulation of lipid metabolism

A20, another OTU family DUB that has both ubiquitinating and deubiquitinating activities, interacts with ACSL4 in lung cancer cells 149. A20 is upregulated by silencing of small ubiquitin-like modifier (SUMO)-specific protease 1(SENP1), which enhances erastin-induced ferroptosis. In addition, ACSL4 expression is also increased when A20 expression level is increased. However, detailed mechanisms remain to be explored.

### Regulation of NRF2

USP11, a USP family DUB, is responsible for the deubiquitination and stabilization of NRF2 in non-small cell lung cancer cells 150. As a result, the depletion of USP11 leads to the induction of ferroptosis and, more importantly, suppresses tumor cell proliferation 103. This finding suggests that the USP11-NRF2 axis may be a potential target for cancer treatment. Villeneuve et al. reported that USP15 negatively regulates NRF2 by deubiquitinating and stabilizing Keap1 151. Thus, USP15 may be involved in the regulation of ferroptosis by increasing the stability of Keap1 and decreasing NRF2 expression. However, further evidence is needed to confirm this hypothesis.

## Implications for targeting ubiquitin enzymes and ferroptosis

Ferroptosis-based therapy has shown promising results in experimental cancer models. An increasing number of studies have shown that inducing ferroptosis in cancer cells might become a new avenue for clinical treatment 11. Unfortunately, owing to the lack of available drug candidates and the varying sensitivity of different tumor types to ferroptosis, patient populations with cancer have not been able to receive full benefits from ferroptosis-inducing agents 152. E3s and DUBs play important roles in regulating cellular ferroptosis sensitivity. On the one hand, E3s and DUBs may be promising predictive biomarkers. On the other hand, the development of new small molecules targeting ubiquitination pathways may considerably accelerate the clinical therapeutic application of ferroptosis. Ubiquitin enzymes can be modulated by anti-tumor drugs, including inhibitors and agonists, proteolysis-targeting chimeras (PROTACs), and molecular glues 145, 153. Significant development of E3 and DUB inhibitors has been witnessed over the past 20 years, with a number of small molecules targeting E3 or DUBs that have come into preclinical use and have been undertaken by industry groups for optimization 154. PROTAC is composed of three parts: an E3 ligand, a protein of interest (POI) ligand, and a linker 155. This bifunctional molecule binds to E3 and POI via two ligands, thus promoting the ubiquitination and proteasomal degradation of POI. Molecular glues are small molecules that can induce a novel association between ubiquitin enzymes and substrates, thereby degrading the substrates 153, 156, 157. However, considering the complexity of ubiquitination regulation, a number of challenges need to be overcome to bring ubiquitin-based proferroptotic therapy into clinical settings. Among these, the specificity and potential adverse effects resulting from the enzymatically promiscuous nature of ubiquitin enzymes must be considered. In addition, ferroptosis occurs not only in tumor cells but also in other cells in the TME, such as TAMs and CD8^+^ T cells; ferroptosis of immune cells sometimes promotes tumor progression 158. Therefore, a deeper understanding of E3s- or DUB-mediated ferroptosis regulation might help to identify the most suitable ubiquitin-based proferroptotic therapies.

## Concluding remarks

Ferroptosis is a new type of cell death that is caused by iron-dependent lipid peroxidation. Although ferroptosis induction is expected to kill tumor cells, it also plays a dual role in cancer progression by targeting different cells in the TME or different action mechanisms. This phenomenon highlights the importance of uncovering the regulatory network of ferroptosis. Ubiquitination is a post-transcriptional modification process that regulates a series of cellular processes by ubiquitinating or deubiquitinating substrates. Abnormalities in enzymes of the ubiquitin system are associated with cancer development and treatment. These enzymes interact with ferroptosis-related proteins and mediate cellular sensitivity to ferroptosis. Recent studies have begun to provide an outline of the collective impact of E3s and DUBs on ferroptosis. However, due to the complexity of ubiquitin enzyme regulation, concrete mechanisms are still enigmatic, including the type of polyubiquitination, ubiquitination site, or other mechanisms independent of their ligase activities. Moreover, our understanding of how E3s and DUBs regulate novel ferroptosis-regulating proteins, such as FSP1, DHODH, and GCH1, remains very limited. Clarifying these questions will drive the development of E3 or DUB inhibitor discovery programs targeting ferroptosis. In general, ubiquitin system enzymes, including E3s and DUBs, play key regulatory roles in ferroptosis; thus, expanding our knowledge of this regulation will lead to innovative strategies for cancer therapy.

## Figures and Tables

**Table 1:** Ferroptosis-related proteins regulated by E3 ubiquitin ligases

E3 ubiquitin ligase type	E3 ubiquitin ligase	Target	Effect on ferroptosis	Reference
RING	TRIM26	SLC7A11	Promote	64
RING	PRC1	SLC7A11	Unclear	66
RING	MITOL	Chac1	Inhibit	71
RING	TRIM46	GPX4	Promote	73
RING	MIB2	GPX4	Promote	74
RING	β-TrCP	TFRC	Inhibit	24
RING	RNF139	HMOX1	Unclear	87
RING	SCF^FBXL5^ complex	IRP2	Unclear	101, 102
RING	FBXO10	ACSL4	Inhibit	105
RING	FBW7	SCD1	Promote	107
RING	MDM2-MDMX complex	PPARα	Promote	110
RING	Keap1-CUL3-RBX1 complex	NRF2	Unclear	113
RING	TRIM21	p62	Promote	117
RING	CRL2-KLHDC3 complex	p14^ARF^	Inhibit	119
RING	MIB1	NRF2	Promote	25
RING	FBXW7	VDAC3	Inhibit	122
RING	SCF^β-TRCP^ complex	YAP, TAZ	Unclear	125, 126
RING	SCF^SKP2^ complex	YAP	Unclear	130
RING	CRL4^DCAF8^ complex	LSH	Promote	134
HECT	NEDD4	VDAC2, VDAC3	Inhibit	26
HECT	HUWE1	TFRC	Inhibit	79
HECT	NEDD4L	Lactotransferrin	Inhibit	80
HECT	SIAH2	HMOX1, NRF2, GPX4	Inhibit	85, 86
HECT	HERC2	NCOA4	Unclear	92
RBR	RNF217	Ferroportin	Unclear	96

**Table 2:** Ferroptosis-related proteins regulated by deubiquitinating enzymes

Deubiquitinating enzyme type	Deubiquitinating enzyme	Target	Effect on ferroptosis	Reference
USP	USP7	SLC7A11	Promote	139
USP	USP7	p53, TFRC	Promote	159
USP	USP7	hnRNPA1	Inhibit	160
USP	USP11	NRF2	Inhibit	150
USP	USP11	Beclin1	Promote	161
USP	USP14	Beclin1	Inhibit	148
USP	USP14	IL-6, NRF2	Promote	162
USP	USP14	NCOA4	Promote	147
USP	USP22	SLC7A11	Inhibit	163
USP	USP35	Ferroportin	Inhibit	94
OTU	OTUB1	SLC7A11	Inhibit	141
OTU	OTUD1	IRP2, TFRC	Promote	146
OTU	A20	ACSL4, SLC7A11	Promote	149
UCH	BAP1	SLC7A11	Promote	66, 137, 138
UCH	BAP1	IP3R	Promote	164

## Abbreviations

ACSL4: acyl-CoA synthetase long-chain family member 4
AGER: advanced glycosylation end product-specific receptor
BAP1: BRCA1-associated protein 1
Chac1: ChaC glutathione-specific γ-glutamylcyclotransferase 1
CRL: Cullin-RING ligase
DAMP: damage-associated molecular pattern
E3: E3 ubiquitin ligase
FBW7: F-box and WD repeat domain-containing 7
FBXL5: F-box and leucine-rich repeat protein 5
FBXO10: F-box only protein 10
FBXO38: F-box protein 38
FBXW7: F-box and WD repeat domain containing 7
FIN: ferroptosis inducer
FPN: ferroportin
GPX4: glutathione peroxidase 4
GSH: glutathione
HECT: homologous to the E6-AP carboxyl terminus
HMOX1: heme oxygenase-1
IRP2: iron regulatory protein 2
JAMM: Jad1/Pad/Mpn domain-containing metalloenzyme
Keap1: Kelch-like ECH-associated protein 1
LSH: lymphoid-specific helicase
MDM2: murine double minute 2
MDMX: murine double minute X
MIB2: mind bomb 2
MINDY: motif interacting with Ub-containing novel DUB family
MITOL: mitochondrial ubiquitin ligase
MJD: Machado-Joseph disease domain protease
NCOA4: nuclear receptor coactivator 4
NEDD4: neuronal precursor cell-expressed developmentally downregulated 4
NRF2: nuclear factor erythroid 2-related factor 2
OTU: ovarian tumor protease
p14^ARF^: p14 alternate reading frame
PDAC: pancreatic ductal adenocarcinoma
PdPT: Palladium pyrithione complex
RBR: RING1-between-RING2
RING: really interesting new gene
RNF: **ri**ng finger protein
ROS: reactive oxygen species
SCD1: stearoyl-CoA desaturase
SCF: Skp1-Cul1-F-box
SENP1: Small ubiquitin-like modifier (SUMO)-specific protease 1
SIAH2: seven in absentia homolog 2
SLC7A11: solute carrier family 7 member 11
TAM: tumor-associated macrophage
TAZ: transcriptional coactivator with PDZ-binding motif
TFRC: transferrin receptor
TME: tumor microenvironment
Treg: regulatory T cell
TRIM: tripartite motif
UCH: Ub C-terminal hydrolase
USP: ubiquitin-specific protease
VDAC: voltage-dependent anion channel
YAP: Yes-associated protein
ZUFSP: zinc-finger and UFSP domain protein

## References

[1] Dixon SJ, Lemberg KM, Lamprecht MR, Skouta R, Zaitsev EM, Gleason CE (2012). Ferroptosis: an iron-dependent form of nonapoptotic cell death. Cell.

[2] Shen Z, Song J, Yung BC, Zhou Z, Wu A, Chen X (2018). Emerging Strategies of Cancer Therapy Based on Ferroptosis. Advanced materials (Deerfield Beach, Fla).

[3] Liang C, Zhang X, Yang M, Dong X (2019). Recent Progress in Ferroptosis Inducers for Cancer Therapy. Advanced materials (Deerfield Beach, Fla).

[4] Viswanathan VS, Ryan MJ, Dhruv HD, Gill S, Eichhoff OM, Seashore-Ludlow B (2017). Dependency of a therapy-resistant state of cancer cells on a lipid peroxidase pathway. Nature.

[5] Tsoi J, Robert L, Paraiso K, Galvan C, Sheu KM, Lay J (2018). Multi-stage Differentiation Defines Melanoma Subtypes with Differential Vulnerability to Drug-Induced Iron-Dependent Oxidative Stress. Cancer cell.

[6] Hassannia B, Wiernicki B, Ingold I, Qu F, Van Herck S, Tyurina YY (2018). Nano-targeted induction of dual ferroptotic mechanisms eradicates high-risk neuroblastoma. The Journal of clinical investigation.

[7] Hangauer MJ, Viswanathan VS, Ryan MJ, Bole D, Eaton JK, Matov A (2017). Drug-tolerant persister cancer cells are vulnerable to GPX4 inhibition. Nature.

[8] Hirschhorn T, Stockwell BR (2019). The development of the concept of ferroptosis. Free radical biology & medicine.

[9] Stockwell BR, Friedmann Angeli JP, Bayir H, Bush AI, Conrad M, Dixon SJ (2017). Ferroptosis: A Regulated Cell Death Nexus Linking Metabolism, Redox Biology, and Disease. Cell.

[10] Wu Y, Zhang S, Gong X, Tam S, Xiao D, Liu S (2020). The epigenetic regulators and metabolic changes in ferroptosis-associated cancer progression. Molecular cancer.

[11] Wang H, Cheng Y, Mao C, Liu S, Xiao D, Huang J (2021). Emerging mechanisms and targeted therapy of ferroptosis in cancer. Mol Ther.

[12] Dai C, Chen X, Li J, Comish P, Kang R, Tang D (2020). Transcription factors in ferroptotic cell death. Cancer gene therapy.

[13] Tang D, Chen X, Kang R, Kroemer G (2021). Ferroptosis: molecular mechanisms and health implications. Cell Res.

[14] Ciechanover A (2003). The ubiquitin proteolytic system and pathogenesis of human diseases: a novel platform for mechanism-based drug targeting. Biochem Soc Trans.

[15] Popovic D, Vucic D, Dikic I (2014). Ubiquitination in disease pathogenesis and treatment. Nat Med.

[16] Schwertman P, Bekker-Jensen S, Mailand N (2016). Regulation of DNA double-strand break repair by ubiquitin and ubiquitin-like modifiers. Nat Rev Mol Cell Biol.

[17] Roberts JZ, Crawford N, Longley DB (2022). The role of Ubiquitination in Apoptosis and Necroptosis. Cell Death Differ.

[18] van Wijk SJ, Fulda S, Dikic I, Heilemann M (2019). Visualizing ubiquitination in mammalian cells. EMBO Rep.

[19] Song L, Luo ZQ (2019). Post-translational regulation of ubiquitin signaling. J Cell Biol.

[20] Harrigan JA, Jacq X, Martin NM, Jackson SP (2018). Deubiquitylating enzymes and drug discovery: emerging opportunities. Nat Rev Drug Discov.

[21] Wang D, Xu C, Yang W, Chen J, Ou Y, Guan Y (2022). E3 ligase RNF167 and deubiquitinase STAMBPL1 modulate mTOR and cancer progression. Molecular cell.

[22] Ren C, Han X, Lu C, Yang T, Qiao P, Sun Y (2022). Ubiquitination of NF-κB p65 by FBXW2 suppresses breast cancer stemness, tumorigenesis, and paclitaxel resistance. Cell death and differentiation.

[23] Yang WL, Wang J, Chan CH, Lee SW, Campos AD, Lamothe B (2009). The E3 ligase TRAF6 regulates Akt ubiquitination and activation. Science (New York, NY).

[24] Guo S, Chen Y, Xue X, Yang Y, Wang Y, Qiu S (2021). TRIB2 desensitizes ferroptosis via βTrCP-mediated TFRC ubiquitiantion in liver cancer cells. Cell death discovery.

[25] Wang H, Huang Q, Xia J, Cheng S, Pei D, Zhang X (2022). The E3 Ligase MIB1 Promotes Proteasomal Degradation of NRF2 and Sensitizes Lung Cancer Cells to Ferroptosis. Mol Cancer Res.

[26] Yang Y, Luo M, Zhang K, Zhang J, Gao T, Connell DO (2020). Nedd4 ubiquitylates VDAC2/3 to suppress erastin-induced ferroptosis in melanoma. Nat Commun.

[27] Doll S, Proneth B, Tyurina YY, Panzilius E, Kobayashi S, Ingold I (2017). ACSL4 dictates ferroptosis sensitivity by shaping cellular lipid composition. Nat Chem Biol.

[28] Dixon SJ, Winter GE, Musavi LS, Lee ED, Snijder B, Rebsamen M (2015). Human Haploid Cell Genetics Reveals Roles for Lipid Metabolism Genes in Nonapoptotic Cell Death. ACS Chem Biol.

[29] Hassannia B, Vandenabeele P, Vanden Berghe T (2019). Targeting Ferroptosis to Iron Out Cancer. Cancer Cell.

[30] Yang WS, Kim KJ, Gaschler MM, Patel M, Shchepinov MS, Stockwell BR (2016). Peroxidation of polyunsaturated fatty acids by lipoxygenases drives ferroptosis. Proc Natl Acad Sci U S A.

[31] Yang WS, SriRamaratnam R, Welsch ME, Shimada K, Skouta R, Viswanathan VS (2014). Regulation of ferroptotic cancer cell death by GPX4. Cell.

[32] Jiang X, Stockwell BR, Conrad M (2021). Ferroptosis: mechanisms, biology and role in disease. Nat Rev Mol Cell Biol.

[33] Doll S, Freitas FP, Shah R, Aldrovandi M, da Silva MC, Ingold I (2019). FSP1 is a glutathione-independent ferroptosis suppressor. Nature.

[34] Bersuker K, Hendricks JM, Li Z, Magtanong L, Ford B, Tang PH (2019). The CoQ oxidoreductase FSP1 acts parallel to GPX4 to inhibit ferroptosis. Nature.

[35] Mao C, Liu X, Zhang Y, Lei G, Yan Y, Lee H (2021). DHODH-mediated ferroptosis defence is a targetable vulnerability in cancer. Nature.

[36] Kraft VAN, Bezjian CT, Pfeiffer S, Ringelstetter L, Muller C, Zandkarimi F (2020). GTP Cyclohydrolase 1/Tetrahydrobiopterin Counteract Ferroptosis through Lipid Remodeling. ACS Cent Sci.

[37] Soula M, Weber RA, Zilka O, Alwaseem H, La K, Yen F (2020). Metabolic determinants of cancer cell sensitivity to canonical ferroptosis inducers. Nat Chem Biol.

[38] Yan HF, Zou T, Tuo QZ, Xu S, Li H, Belaidi AA (2021). Ferroptosis: mechanisms and links with diseases. Signal Transduct Target Ther.

[39] Lei G, Zhuang L, Gan B (2022). Targeting ferroptosis as a vulnerability in cancer. Nat Rev Cancer.

[40] Zhang C, Liu X, Jin S, Chen Y, Guo R (2022). Ferroptosis in cancer therapy: a novel approach to reversing drug resistance. Molecular cancer.

[41] Daher B, Parks SK, Durivault J, Cormerais Y, Baidarjad H, Tambutte E (2019). Genetic Ablation of the Cystine Transporter xCT in PDAC Cells Inhibits mTORC1, Growth, Survival, and Tumor Formation via Nutrient and Oxidative Stresses. Cancer research.

[42] Yu H, Yang C, Jian L, Guo S, Chen R, Li K (2019). Sulfasalazine-induced ferroptosis in breast cancer cells is reduced by the inhibitory effect of estrogen receptor on the transferrin receptor. Oncology reports.

[43] Dixon SJ, Patel DN, Welsch M, Skouta R, Lee ED, Hayano M (2014). Pharmacological inhibition of cystine-glutamate exchange induces endoplasmic reticulum stress and ferroptosis. eLife.

[44] Louandre C, Marcq I, Bouhlal H, Lachaier E, Godin C, Saidak Z (2015). The retinoblastoma (Rb) protein regulates ferroptosis induced by sorafenib in human hepatocellular carcinoma cells. Cancer letters.

[45] Ghoochani A, Hsu EC, Aslan M, Rice MA, Nguyen HM, Brooks JD (2021). Ferroptosis Inducers Are a Novel Therapeutic Approach for Advanced Prostate Cancer. Cancer research.

[46] Zhang Y, Tan H, Daniels JD, Zandkarimi F, Liu H, Brown LM (2019). Imidazole Ketone Erastin Induces Ferroptosis and Slows Tumor Growth in a Mouse Lymphoma Model. Cell chemical biology.

[47] Woo JH, Shimoni Y, Yang WS, Subramaniam P, Iyer A, Nicoletti P (2015). Elucidating Compound Mechanism of Action by Network Perturbation Analysis. Cell.

[48] Fan F, Liu P, Bao R, Chen J, Zhou M, Mo Z (2021). A Dual PI3K/HDAC Inhibitor Induces Immunogenic Ferroptosis to Potentiate Cancer Immune Checkpoint Therapy. Cancer research.

[49] Efimova I, Catanzaro E, Van der Meeren L, Turubanova VD, Hammad H, Mishchenko TA (2020). Vaccination with early ferroptotic cancer cells induces efficient antitumor immunity. Journal for immunotherapy of cancer.

[50] Gu Z, Liu T, Liu C, Yang Y, Tang J, Song H (2021). Ferroptosis-Strengthened Metabolic and Inflammatory Regulation of Tumor-Associated Macrophages Provokes Potent Tumoricidal Activities. Nano letters.

[51] Dai E, Han L, Liu J, Xie Y, Zeh HJ, Kang R (2020). Ferroptotic damage promotes pancreatic tumorigenesis through a TMEM173/STING-dependent DNA sensor pathway. Nature communications.

[52] Dai E, Han L, Liu J, Xie Y, Kroemer G, Klionsky DJ (2020). Autophagy-dependent ferroptosis drives tumor-associated macrophage polarization via release and uptake of oncogenic KRAS protein. Autophagy.

[53] Ma X, Xiao L, Liu L, Ye L, Su P, Bi E (2021). CD36-mediated ferroptosis dampens intratumoral CD8(+) T cell effector function and impairs their antitumor ability. Cell metabolism.

[54] Lang X, Green MD, Wang W, Yu J, Choi JE, Jiang L (2019). Radiotherapy and Immunotherapy Promote Tumoral Lipid Oxidation and Ferroptosis via Synergistic Repression of SLC7A11. Cancer Discov.

[55] Lei G, Zhang Y, Koppula P, Liu X, Zhang J, Lin SH (2020). The role of ferroptosis in ionizing radiation-induced cell death and tumor suppression. Cell research.

[56] Ye LF, Chaudhary KR, Zandkarimi F, Harken AD, Kinslow CJ, Upadhyayula PS (2020). Radiation-Induced Lipid Peroxidation Triggers Ferroptosis and Synergizes with Ferroptosis Inducers. ACS Chem Biol.

[57] Guo J, Xu B, Han Q, Zhou H, Xia Y, Gong C (2018). Ferroptosis: A Novel Anti-tumor Action for Cisplatin. Cancer research and treatment.

[58] Mou Y, Wang J, Wu J, He D, Zhang C, Duan C (2019). Ferroptosis, a new form of cell death: opportunities and challenges in cancer. Journal of hematology & oncology.

[59] Roh JL, Kim EH, Jang HJ, Park JY, Shin D (2016). Induction of ferroptotic cell death for overcoming cisplatin resistance of head and neck cancer. Cancer letters.

[60] Wang W, Green M, Choi JE, Gijón M, Kennedy PD, Johnson JK (2019). CD8(+) T cells regulate tumour ferroptosis during cancer immunotherapy. Nature.

[61] Jiang Q, Wang K, Zhang X, Ouyang B, Liu H, Pang Z (2020). Platelet Membrane-Camouflaged Magnetic Nanoparticles for Ferroptosis-Enhanced Cancer Immunotherapy. Small (Weinheim an der Bergstrasse, Germany).

[62] Lin W, Wang C, Liu G, Bi C, Wang X, Zhou Q (2020). SLC7A11/xCT in cancer: biological functions and therapeutic implications. American journal of cancer research.

[63] Koppula P, Zhang Y, Zhuang L, Gan B (2018). Amino acid transporter SLC7A11/xCT at the crossroads of regulating redox homeostasis and nutrient dependency of cancer. Cancer communications (London, England).

[64] Zhu Y, Zhang C, Huang M, Lin J, Fan X, Ni T (2021). TRIM26 Induces Ferroptosis to Inhibit Hepatic Stellate Cell Activation and Mitigate Liver Fibrosis Through Mediating SLC7A11 Ubiquitination. Front Cell Dev Biol.

[65] Kallin EM, Cao R, Jothi R, Xia K, Cui K, Zhao K (2009). Genome-wide uH2A localization analysis highlights Bmi1-dependent deposition of the mark at repressed genes. PLoS Genet.

[66] Zhang Y, Koppula P, Gan B (2019). Regulation of H2A ubiquitination and SLC7A11 expression by BAP1 and PRC1. Cell Cycle.

[67] Chen X, Li J, Kang R, Klionsky DJ, Tang D (2021). Ferroptosis: machinery and regulation. Autophagy.

[68] Ursini F, Maiorino M (2020). Lipid peroxidation and ferroptosis: The role of GSH and GPx4. Free radical biology & medicine.

[69] Crawford RR, Prescott ET, Sylvester CF, Higdon AN, Shan J, Kilberg MS (2015). Human CHAC1 Protein Degrades Glutathione, and mRNA Induction Is Regulated by the Transcription Factors ATF4 and ATF3 and a Bipartite ATF/CRE Regulatory Element. J Biol Chem.

[70] Wang N, Zeng GZ, Yin JL, Bian ZX (2019). Artesunate activates the ATF4-CHOP-CHAC1 pathway and affects ferroptosis in Burkitt's Lymphoma. Biochemical and biophysical research communications.

[71] Kitakata H, Endo J, Matsushima H, Yamamoto S, Ikura H, Hirai A (2021). MITOL/MARCH5 determines the susceptibility of cardiomyocytes to doxorubicin-induced ferroptosis by regulating GSH homeostasis. J Mol Cell Cardiol.

[72] Stockwell BR, Jiang X, Gu W (2020). Emerging Mechanisms and Disease Relevance of Ferroptosis. Trends in cell biology.

[73] Zhang J, Qiu Q, Wang H, Chen C, Luo D (2021). TRIM46 contributes to high glucose-induced ferroptosis and cell growth inhibition in human retinal capillary endothelial cells by facilitating GPX4 ubiquitination. Exp Cell Res.

[74] Zhao L, Gong H, Huang H, Tuerhong G, Xia H (2021). Participation of Mind Bomb-2 in Sevoflurane Anesthesia Induces Cognitive Impairment in Aged Mice via Modulating Ferroptosis. ACS Chem Neurosci.

[75] Chen X, Yu C, Kang R, Tang D (2020). Iron Metabolism in Ferroptosis. Frontiers in cell and developmental biology.

[76] Kawabata H (2019). Transferrin and transferrin receptors update. Free radical biology & medicine.

[77] Gao M, Monian P, Quadri N, Ramasamy R, Jiang X (2015). Glutaminolysis and Transferrin Regulate Ferroptosis. Molecular cell.

[78] Lu Y, Yang Q, Su Y, Ji Y, Li G, Yang X (2021). MYCN mediates TFRC-dependent ferroptosis and reveals vulnerabilities in neuroblastoma. Cell death & disease.

[79] Wu Y, Jiao H, Yue Y, He K, Jin Y, Zhang J (2022). Ubiquitin ligase E3 HUWE1/MULE targets transferrin receptor for degradation and suppresses ferroptosis in acute liver injury. Cell Death Differ.

[80] Wang Y, Liu Y, Liu J, Kang R, Tang D (2020). NEDD4L-mediated LTF protein degradation limits ferroptosis. Biochem Biophys Res Commun.

[81] Chang LC, Chiang SK, Chen SE, Yu YL, Chou RH, Chang WC (2018). Heme oxygenase-1 mediates BAY 11-7085 induced ferroptosis. Cancer letters.

[82] Kwon MY, Park E, Lee SJ, Chung SW (2015). Heme oxygenase-1 accelerates erastin-induced ferroptotic cell death. Oncotarget.

[83] Sun X, Ou Z, Chen R, Niu X, Chen D, Kang R (2016). Activation of the p62-Keap1-NRF2 pathway protects against ferroptosis in hepatocellular carcinoma cells. Hepatology.

[84] Adedoyin O, Boddu R, Traylor A, Lever JM, Bolisetty S, George JF (2018). Heme oxygenase-1 mitigates ferroptosis in renal proximal tubule cells. American journal of physiology Renal physiology.

[85] Chillappagari S, Belapurkar R, Moller A, Molenda N, Kracht M, Rohrbach S (2020). SIAH2-mediated and organ-specific restriction of HO-1 expression by a dual mechanism. Sci Rep.

[86] Baba K, Morimoto H, Imaoka S (2013). Seven in absentia homolog 2 (Siah2) protein is a regulator of NF-E2-related factor 2 (Nrf2). J Biol Chem.

[87] Lin PH, Lan WM, Chau LY (2013). TRC8 suppresses tumorigenesis through targeting heme oxygenase-1 for ubiquitination and degradation. Oncogene.

[88] Zhang N, Yu X, Xie J, Xu H (2021). New Insights into the Role of Ferritin in Iron Homeostasis and Neurodegenerative Diseases. Molecular neurobiology.

[89] Mancias JD, Wang X, Gygi SP, Harper JW, Kimmelman AC (2014). Quantitative proteomics identifies NCOA4 as the cargo receptor mediating ferritinophagy. Nature.

[90] Gao M, Monian P, Pan Q, Zhang W, Xiang J, Jiang X (2016). Ferroptosis is an autophagic cell death process. Cell research.

[91] Hou W, Xie Y, Song X, Sun X, Lotze MT, Zeh HJ 3rd (2016). Autophagy promotes ferroptosis by degradation of ferritin. Autophagy.

[92] Mancias JD, Pontano Vaites L, Nissim S, Biancur DE, Kim AJ, Wang X (2015). Ferritinophagy via NCOA4 is required for erythropoiesis and is regulated by iron dependent HERC2-mediated proteolysis. eLife.

[93] Yang Q, Liu W, Zhang S, Liu S (2020). The cardinal roles of ferroportin and its partners in controlling cellular iron in and out. Life sciences.

[94] Tang Z, Jiang W, Mao M, Zhao J, Chen J, Cheng N (2021). Deubiquitinase USP35 modulates ferroptosis in lung cancer via targeting ferroportin. Clin Transl Med.

[95] Li Y, Zeng X, Lu D, Yin M, Shan M, Gao Y (2021). Erastin induces ferroptosis via ferroportin-mediated iron accumulation in endometriosis. Human reproduction (Oxford, England).

[96] Jiang L, Wang J, Wang K, Wang H, Wu Q, Yang C (2021). RNF217 regulates iron homeostasis through its E3 ubiquitin ligase activity by modulating ferroportin degradation. Blood.

[97] Anderson GJ, Frazer DM (2017). Current understanding of iron homeostasis. Am J Clin Nutr.

[98] Katsarou A, Pantopoulos K (2020). Basics and principles of cellular and systemic iron homeostasis. Molecular aspects of medicine.

[99] Li Y, Jin C, Shen M, Wang Z, Tan S, Chen A (2020). Iron regulatory protein 2 is required for artemether -mediated anti-hepatic fibrosis through ferroptosis pathway. Free radical biology & medicine.

[100] Terzi EM, Sviderskiy VO, Alvarez SW, Whiten GC, Possemato R (2021). Iron-sulfur cluster deficiency can be sensed by IRP2 and regulates iron homeostasis and sensitivity to ferroptosis independent of IRP1 and FBXL5. Science advances.

[101] Vashisht AA, Zumbrennen KB, Huang X, Powers DN, Durazo A, Sun D (2009). Control of iron homeostasis by an iron-regulated ubiquitin ligase. Science.

[102] Salahudeen AA, Thompson JW, Ruiz JC, Ma HW, Kinch LN, Li Q (2009). An E3 ligase possessing an iron-responsive hemerythrin domain is a regulator of iron homeostasis. Science.

[103] Muto Y, Nishiyama M, Nita A, Moroishi T, Nakayama KI (2017). Essential role of FBXL5-mediated cellular iron homeostasis in maintenance of hematopoietic stem cells. Nat Commun.

[104] Li D, Li Y (2020). The interaction between ferroptosis and lipid metabolism in cancer. Signal transduction and targeted therapy.

[105] Bao Z, Liu Y, Chen B, Miao Z, Tu Y, Li C (2021). Prokineticin-2 prevents neuronal cell deaths in a model of traumatic brain injury. Nat Commun.

[106] Tesfay L, Paul BT, Konstorum A, Deng Z, Cox AO, Lee J (2019). Stearoyl-CoA Desaturase 1 Protects Ovarian Cancer Cells from Ferroptotic Cell Death. Cancer research.

[107] Ye Z, Zhuo Q, Hu Q, Xu X, Mengqi L, Zhang Z (2021). FBW7-NRA41-SCD1 axis synchronously regulates apoptosis and ferroptosis in pancreatic cancer cells. Redox Biol.

[108] Karni-Schmidt O, Lokshin M, Prives C (2016). The Roles of MDM2 and MDMX in Cancer. Annual review of pathology.

[109] Wade M, Li YC, Wahl GM (2013). MDM2, MDMX and p53 in oncogenesis and cancer therapy. Nature reviews Cancer.

[110] Venkatesh D, O'Brien NA, Zandkarimi F, Tong DR, Stokes ME, Dunn DE (2020). MDM2 and MDMX promote ferroptosis by PPARα-mediated lipid remodeling. Genes Dev.

[111] Dodson M, Castro-Portuguez R, Zhang DD (2019). NRF2 plays a critical role in mitigating lipid peroxidation and ferroptosis. Redox biology.

[112] Fan Z, Wirth AK, Chen D, Wruck CJ, Rauh M, Buchfelder M (2017). Nrf2-Keap1 pathway promotes cell proliferation and diminishes ferroptosis. Oncogenesis.

[113] Baird L, Yamamoto M (2020). The Molecular Mechanisms Regulating the KEAP1-NRF2 Pathway. Mol Cell Biol.

[114] Yamamoto M, Kensler TW, Motohashi H (2018). The KEAP1-NRF2 System: a Thiol-Based Sensor-Effector Apparatus for Maintaining Redox Homeostasis. Physiological reviews.

[115] Silva-Islas CA, Maldonado PD (2018). Canonical and non-canonical mechanisms of Nrf2 activation. Pharmacological research.

[116] Pan JA, Sun Y, Jiang YP, Bott AJ, Jaber N, Dou Z (2016). TRIM21 Ubiquitylates SQSTM1/p62 and Suppresses Protein Sequestration to Regulate Redox Homeostasis. Molecular cell.

[117] Hou K, Shen J, Yan J, Zhai C, Zhang J, Pan JA (2021). Loss of TRIM21 alleviates cardiotoxicity by suppressing ferroptosis induced by the chemotherapeutic agent doxorubicin. EBioMedicine.

[118] Chen D, Tavana O, Chu B, Erber L, Chen Y, Baer R (2017). NRF2 Is a Major Target of ARF in p53-Independent Tumor Suppression. Molecular cell.

[119] Zhang P, Gao K, Zhang L, Sun H, Zhao X, Liu Y (2021). CRL2-KLHDC3 E3 ubiquitin ligase complex suppresses ferroptosis through promoting p14(ARF) degradation. Cell Death Differ.

[120] Rosencrans WM, Rajendran M, Bezrukov SM, Rostovtseva TK (2021). VDAC regulation of mitochondrial calcium flux: From channel biophysics to disease. Cell calcium.

[121] Zhao Y, Li Y, Zhang R, Wang F, Wang T, Jiao Y (2020). The Role of Erastin in Ferroptosis and Its Prospects in Cancer Therapy. OncoTargets and therapy.

[122] Zhu T, Liu B, Wu D, Xu G, Fan Y (2021). Autophagy Regulates VDAC3 Ubiquitination by FBXW7 to Promote Erastin-Induced Ferroptosis in Acute Lymphoblastic Leukemia. Frontiers in cell and developmental biology.

[123] Sun T, Chi JT (2021). Regulation of ferroptosis in cancer cells by YAP/TAZ and Hippo pathways: The therapeutic implications. Genes & diseases.

[124] Ma S, Meng Z, Chen R, Guan KL (2019). The Hippo Pathway: Biology and Pathophysiology. Annu Rev Biochem.

[125] Liu CY, Zha ZY, Zhou X, Zhang H, Huang W, Zhao D (2010). The hippo tumor pathway promotes TAZ degradation by phosphorylating a phosphodegron and recruiting the SCF{beta}-TrCP E3 ligase. J Biol Chem.

[126] Zhao B, Li L, Tumaneng K, Wang CY, Guan KL (2010). A coordinated phosphorylation by Lats and CK1 regulates YAP stability through SCF(beta-TRCP). Genes Dev.

[127] Wu J, Minikes AM, Gao M, Bian H, Li Y, Stockwell BR (2019). Intercellular interaction dictates cancer cell ferroptosis via NF2-YAP signalling. Nature.

[128] Yang WH, Ding CC, Sun T, Rupprecht G, Lin CC, Hsu D (2019). The Hippo Pathway Effector TAZ Regulates Ferroptosis in Renal Cell Carcinoma. Cell reports.

[129] Yang WH, Huang Z, Wu J, Ding CC, Murphy SK, Chi JT (2020). A TAZ-ANGPTL4-NOX2 Axis Regulates Ferroptotic Cell Death and Chemoresistance in Epithelial Ovarian Cancer. Molecular cancer research: MCR.

[130] Yao F, Zhou Z, Kim J, Hang Q, Xiao Z, Ton BN (2018). SKP2- and OTUD1-regulated non-proteolytic ubiquitination of YAP promotes YAP nuclear localization and activity. Nat Commun.

[131] Yang WH, Lin CC, Wu J, Chao PY, Chen K, Chen PH (2021). The Hippo Pathway Effector YAP Promotes Ferroptosis via the E3 Ligase SKP2. Molecular cancer research: MCR.

[132] Chen X, Li Y, Rubio K, Deng B, Li Y, Tang Q (2022). Lymphoid-specific helicase in epigenetics, DNA repair and cancer. British journal of cancer.

[133] Jiang Y, Mao C, Yang R, Yan B, Shi Y, Liu X (2017). EGLN1/c-Myc Induced Lymphoid-Specific Helicase Inhibits Ferroptosis through Lipid Metabolic Gene Expression Changes. Theranostics.

[134] Huang D, Li Q, Sun X, Sun X, Tang Y, Qu Y (2021). CRL4(DCAF8) dependent opposing stability control over the chromatin remodeler LSH orchestrates epigenetic dynamics in ferroptosis. Cell Death Differ.

[135] Lange SM, Armstrong LA, Kulathu Y (2022). Deubiquitinases: From mechanisms to their inhibition by small molecules. Mol Cell.

[136] Cheng J, Guo J, North BJ, Wang B, Cui CP, Li H (2019). Functional analysis of deubiquitylating enzymes in tumorigenesis and development. Biochim Biophys Acta Rev Cancer.

[137] Zhang Y, Shi J, Liu X, Feng L, Gong Z, Koppula P (2018). BAP1 links metabolic regulation of ferroptosis to tumour suppression. Nat Cell Biol.

[138] Zhang Y, Zhuang L, Gan B (2019). BAP1 suppresses tumor development by inducing ferroptosis upon SLC7A11 repression. Mol Cell Oncol.

[139] Wang Y, Yang L, Zhang X, Cui W, Liu Y, Sun QR (2019). Epigenetic regulation of ferroptosis by H2B monoubiquitination and p53. EMBO Rep.

[140] Liu T, Jiang L, Tavana O, Gu W (2019). The Deubiquitylase OTUB1 Mediates Ferroptosis via Stabilization of SLC7A11. Cancer Res.

[141] Zhao X, Zhou M, Yang Y, Luo M (2021). The ubiquitin hydrolase OTUB1 promotes glioma cell stemness via suppressing ferroptosis through stabilizing SLC7A11 protein. Bioengineered.

[142] Chen S, Bu D, Zhu J, Yue T, Guo S, Wang X (2021). Endogenous hydrogen sulfide regulates xCT stability through persulfidation of OTUB1 at cysteine 91 in colon cancer cells. Neoplasia.

[143] Wang S, Wang Y, Li Q, Li X, Feng X (2022). A novel circular RNA confers trastuzumab resistance in human epidermal growth factor receptor 2-positive breast cancer through regulating ferroptosis. Environ Toxicol.

[144] Liang X, Hu C, Han M, Liu C, Sun X, Yu K (2022). Solasonine Inhibits Pancreatic Cancer Progression With Involvement of Ferroptosis Induction. Front Oncol.

[145] Yang L, Chen X, Yang Q, Chen J, Huang Q, Yao L (2020). Broad Spectrum Deubiquitinase Inhibition Induces Both Apoptosis and Ferroptosis in Cancer Cells. Front Oncol.

[146] Song J, Liu T, Yin Y, Zhao W, Lin Z, Yin Y (2021). The deubiquitinase OTUD1 enhances iron transport and potentiates host antitumor immunity. EMBO Rep.

[147] Li C, Sun G, Chen B, Xu L, Ye Y, He J (2021). Nuclear receptor coactivator 4-mediated ferritinophagy contributes to cerebral ischemia-induced ferroptosis in ischemic stroke. Pharmacol Res.

[148] Tsai Y, Xia C, Sun Z (2020). The Inhibitory Effect of 6-Gingerol on Ubiquitin-Specific Peptidase 14 Enhances Autophagy-Dependent Ferroptosis and Anti-Tumor in vivo and in vitro. Front Pharmacol.

[149] Gao C, Xiao F, Zhang L, Sun Y, Wang L, Liu X (2022). SENP1 inhibition suppresses the growth of lung cancer cells through activation of A20-mediated ferroptosis. Ann Transl Med.

[150] Meng C, Zhan J, Chen D, Shao G, Zhang H, Gu W (2021). The deubiquitinase USP11 regulates cell proliferation and ferroptotic cell death via stabilization of NRF2 USP11 deubiquitinates and stabilizes NRF2. Oncogene.

[151] Villeneuve NF, Tian W, Wu T, Sun Z, Lau A, Chapman E (2013). USP15 negatively regulates Nrf2 through deubiquitination of Keap1. Mol Cell.

[152] Chen X, Kang R, Kroemer G, Tang D (2021). Broadening horizons: the role of ferroptosis in cancer. Nature reviews Clinical oncology.

[153] Ye P, Chi X, Cha JH, Luo S, Yang G, Yan X (2021). Potential of E3 Ubiquitin Ligases in Cancer Immunity: Opportunities and Challenges. Cells.

[154] Schauer NJ, Magin RS, Liu X, Doherty LM, Buhrlage SJ (2020). Advances in Discovering Deubiquitinating Enzyme (DUB) Inhibitors. J Med Chem.

[155] Toure M, Crews CM (2016). Small-Molecule PROTACS: New Approaches to Protein Degradation. Angewandte Chemie (International ed in English).

[156] Dale B, Cheng M, Park KS, Kaniskan H, Xiong Y, Jin J (2021). Advancing targeted protein degradation for cancer therapy. Nature reviews Cancer.

[157] Dong G, Ding Y, He S, Sheng C (2021). Molecular Glues for Targeted Protein Degradation: From Serendipity to Rational Discovery. Journal of medicinal chemistry.

[158] Xu H, Ye D, Ren M, Zhang H, Bi F (2021). Ferroptosis in the tumor microenvironment: perspectives for immunotherapy. Trends in molecular medicine.

[159] Tang LJ, Zhou YJ, Xiong XM, Li NS, Zhang JJ, Luo XJ (2021). Ubiquitin-specific protease 7 promotes ferroptosis via activation of the p53/TfR1 pathway in the rat hearts after ischemia/reperfusion. Free Radic Biol Med.

[160] Zhang H, Deng T, Liu R, Ning T, Yang H, Liu D (2020). CAF secreted miR-522 suppresses ferroptosis and promotes acquired chemo-resistance in gastric cancer. Mol Cancer.

[161] Rong Y, Fan J, Ji C, Wang Z, Ge X, Wang J (2022). USP11 regulates autophagy-dependent ferroptosis after spinal cord ischemia-reperfusion injury by deubiquitinating Beclin 1. Cell Death Differ.

[162] Zhu G, Sui S, Shi F, Wang Q (2022). Inhibition of USP14 suppresses ferroptosis and inflammation in LPS-induced goat mammary epithelial cells through ubiquitylating the IL-6 protein. Hereditas.

[163] Ma S, Sun L, Wu W, Wu J, Sun Z, Ren J (2020). USP22 Protects Against Myocardial Ischemia-Reperfusion Injury via the SIRT1-p53/SLC7A11-Dependent Inhibition of Ferroptosis-Induced Cardiomyocyte Death. Front Physiol.

[164] Ye Y, Li X, Feng G, Ma Y, Ye F, Shen H (2022). 3,3'-Diindolylmethane induces ferroptosis by BAP1-IP3R axis in BGC-823 gastric cancer cells. Anticancer Drugs.

